# Anti-Inflammatory Activity of Chitooligosaccharides *in Vivo*

**DOI:** 10.3390/md8061763

**Published:** 2010-05-28

**Authors:** João C. Fernandes, Humberto Spindola, Vanessa de Sousa, Alice Santos-Silva, Manuela E. Pintado, Francisco Xavier Malcata, João E. Carvalho

**Affiliations:** 1 CBQF/Escola Superior de Biotecnologia, Universidade Católica Portuguesa, Rua Dr. António Bernardino de Almeida, P-4200-072 Porto, Portugal; E-Mails: mmpintado@esb.ucp.pt (M.E.P.); fxmalcata@docente.ismai.pt (F.X.M.); 2 CPQBA/Divisão de Farmacologia e Toxicologia, Universidade Estadual de Campinas, Campinas, São Paulo, Brazil; E-Mails: hmspindola@hotmail.com (H.S.); vanahelena@hotmail.com (V.d.S.); carvalho_je@yahoo.com.br (J.E.C.); 3 Serviço de Bioquímica, Faculdade de Farmácia da Universidade do Porto, Rua Aníbal Cunha, P-4050-047 Porto, Portugal; E-Mail: assilva@ff.up.pt (A.S.-S.); 4 Instituto de Biologia Molecular e Celular (IBMC) da Universidade do Porto, Rua do Campo Alegre, P-4169-007 Porto, Portugal

**Keywords:** chitooligosaccharides, anti-inflammatory, animal model

## Abstract

All the reports to date on the anti-inflammatory activity of chitooligosaccharides (COS) are mostly based on *in vitro* methods. In this work, the anti-inflammatory activity of two COS mixtures is characterized *in vivo* (using balb/c mice), following the carrageenan-induced paw edema method. This is a widely accepted animal model of acute inflammation to evaluate the anti-inflammatory effect of drugs. Our data suggest that COS possess anti-inflammatory activity, which is dependent on dose and, at higher doses, also on the molecular weight. A single dose of 500 mg/kg b.w. weight may be suitable to treat acute inflammation cases; however, further studies are needed to ascertain the effect upon longer inflammation periods as well as studies upon the bioavailability of these compounds.

## 1. Introduction

Chitooligosaccharides (COS) are partially hydrolyzed products of chitosan, a biopolymer composed of β-(1–4)-linked *N*-acetyl-d-glucosamine and deacetylated glucosamine units [1]. Several authors have reported the potential of COS as a therapeutic agent against inflammation. These studies were mainly based on *in vitro* tests [2–4]. It has been suggested that this anti-inflammatory action of COS occurs via down-regulation of transcriptional and translational expression levels of TNF-α, IL-6, iNOS and COX-2 [4–6]; furthermore, it depends on the molecular weight of COS [6]. This preliminary study, therefore, intends to investigate the anti-inflammatory activities of COS mixtures with different molecular weights, by studying its effects *in vivo* upon inflammation induced by carrageenan.

## 2. Results and Discussion

Both COS mixtures administered orally at doses between 50–1,000 mg/kg b.w., didn’t generate any significant change in the autonomic or behavioural responses during the observation period. Therefore, the oral LD_50_ value in mice, for both COS, was found to be above 1,000 mg/kg b.w.

Edema induced by phlogistic agents is a widely accepted model for the evaluation of anti-inflammatory effect of drugs [7]. Carrageenan-induced paw edema is a classical model of acute inflammation (used mainly for testing the nonsteroidal anti-inflammatory drugs, as INN) involving various types of chemical mediators of inflammation such as histamine, serotonin, bradykinin and prostaglandins, in which the involvement of the cyclooxygenase products of arachidonic acid metabolism and the production of reactive oxygen species are well established [8]. Development of edema induced by carrageenan is commonly correlated with the early exudative stage of inflammation, one of the important processes of inflammatory pathology [9]. In the beginning of carrageenan injection, there is sudden elevation of paw volume as consequence of histamine liberation from mastocyte cells [10]. After 1 h the inflammation increases gradually and is elevated during the later 3–6 h. This second phase is mediated by prostaglandins, cyclooxygenase products. Continuity between the two phases is provided by kinins [11,12].

**Figure 1:**
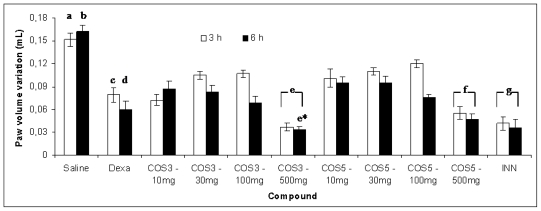
Effect of various doses of both COS, administered orally 60 min prior to injection of carrageenan, on mice paw edema volume (mL), after 3 and 6 h. (Average ± S.E.M.). Legend: (a) statistically different from all other compounds tested (*p* < 0.05), except b; (b) statistically different from all other compounds tested (*p* < 0.05), except a; (c) statistically different (*p* < 0.05) from COS3–500 mg and INN at 3 and 6 h; (d) statistically different from COS3–500 mg and INN at 3 and 6 h; (e) statistically different (*p* < 0.05) from other COS3 concentrations; (e*) statistically different from COS5–500 mg at 6 h (*p* < 0.05); (f) statistically different from other COS5 concentrations; (g) statistically different from COS3 and COS5 concentrations, except 500 mg values (*p* < 0.05).

To demonstrate the validity of the carrageenan-induced paw edema test, mice were administered INN orally as a positive control at a dosage of 10 mg/kg b.w. 1 h before carrageenan injection. As expected, INN significantly (*p* < 0.05) decreased paw edema at 2, 3 and 6 h after carrageenan injection compared to saline, with inhibition levels of 67.92%, 71.61% and 78.79%, respectively (Figure 1). These results demonstrate that INN, a cyclooxygenase inhibitor, exerts an anti-edematous effect during the second phase of paw edema due to the reduction of prostaglandins, which are second phase inflammatory mediators. Simultaneously, mice were administered various doses of COS3 and COS5 (10–500 mg/kg b.w.) orally 1 h before carrageenan administration. All tested concentrations significantly (*p* < 0.05) decreased the paw volume at 3 and 6 h after carrageenan administration compared to vehicle control (Figure 1). Both COS showed higher action at 500 mg/kg, decreasing the paw volume significantly compared to the other tested concentrations. Also, at this concentration the molecular weight proved to play a major role reducing paw volume, since COS3 showed significant (*p* < 0.05) stronger effect −73.42% and 78.13%, than COS5–63.20% and 71.88% at 3 and 6 h, respectively.

**Figure 2:**
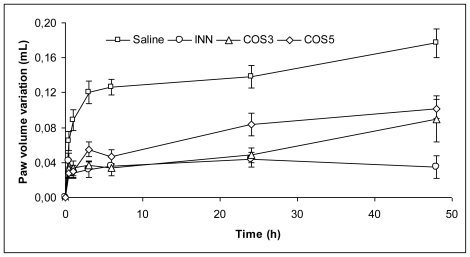
Effect of 500 mg/kg b.w. of both COS, administered orally 60 min prior to injection of carrageenan, on mice paw edema volume (mL), along the time (Average ± S.E.M.).

Overall every dose of COS3 and COS5 tested in this study showed significant reduction of paw edema at 2 h after carrageenan injection, suggesting that COS produces an anti-edematous effect during the second phase, similarly to INN. Therefore, our results suggest that the mechanism of the anti-inflammatory effect of COS may involve the inhibition of the cyclooxygenase pathway; as reported elsewhere [6], COS may exert their anti-inflammatory effect via down-regulation of transcriptional and translational expression levels of COX-2. At this stage, an endpoint was established in order to prevent animals from suffering a severe discomfort resulting from leg ulceration. However, the experiment was extended with the INN group (which showed to be more effective than Dexa as a positive control), and with the most promising COS concentration–500 mg/kg (Figure 2).

COS3 continued to exert anti-inflammatory activity, comparable to INN until the 24 h, while COS5 started losing activity around the 15–h which may be related to a lower level of COS5 in the blood circulation, due to its higher MW and concomitant lower absorption rate at intestinal level [1]; by 48 h, both COS mixtures presented a similar effect (between 43–47%), lower than INN (81.81%) but still significantly different from the negative control (p < 0.05). After euthanasia, no changes were observed in COS3 and COS5 administered mice. Kidneys, liver, stomach, heart and intestines were analyzed for visible alterations and weighted, presenting no differences compared to control mice; however, the group administered with INN, had considerably inflated and heavier stomachs and intestines–which can be associated to the peptic ulcer inducing effect of INN [13].

## 3. Experimental Section

### 3.1. Materials

Chitooligosaccharide mixtures characterized by two distinct average molecular weights 1.2 (COS3)—and 5.3 kDa (COS5)—and possessing a degree of deacetylation in the 80–85% range, were purchased from Nicechem (Shanghai, China). Both compounds were derived from crab shells. All chemicals used in this work were purchased from Sigma-Brazil.

### 3.2. Animals

Balb/c male mice (6 weeks), weighing between 27 and 32 g, were used in the experiments. The animals were purchased from Centro Multidisciplinar para Investigação Biológica na Área da Ciência em Animais de Laboratório–CEMIB at the University of Campinas (UNICAMP), and were kept in polyethylene boxes (n = 6), in a controlled environment—constant temperature (24 ± 2 °C) with a 12 h light-dark cycle and relative humidity of 40–70%. They were kept without food for 24 h before the experiment and water was *ad libitum*. Groups of six mice were used and the studies were carried out in accordance with current guidelines for the veterinary care of laboratory animals [14], and were performed under the consent and surveillance of Unicamp’s Institute of Biology Ethics Committee for Animal Research (1076-1).

### 3.3. Preliminary screening and acute toxicity assessment

Mice were divided into seven groups, each containing six animals. COS mixtures were administered orally, in varying doses (50, 250 and 1,000 mg/kg b.w.) to these animals, using saline solution as a vehicle. A group of animals treated with the vehicle served as control. They were continuously observed for 4 h to detect changes in their autonomic or behavioural responses viz. alertness, spontaneous activity, irritability, pinna reflex, corneal reflex, urination, salivation and piloerection [15]. Any mortality during this period of experimentation and along the following 14 days was also recorded. Based on the results of this preliminary toxicity test, doses of 10, 30, 100 and 500 mg/kg b.w. were chosen to study the anti-inflammatory activity of COS mixtures.

### 3.4. Acute inflammation assessment

Anti-inflammatory activity was studied by carrageenan-induced paw edema method [7]. Mice were divided into eleven groups, each containing six animals. COS mixtures were administered orally in different doses (10, 30, 100 and 500 mg/kg b.w.), 60 min prior to carrageenan injection. Two positive control groups [16] were used: one with 10 mg/kg b.w. of indomethacin (INN–a well-known non-steroidal anti-inflammatory drug), and the other with 1 mg/kg b.w. of dexamethasone (Dexa–a potent synthetic member of the glucocorticoid class of steroid hormones). A group of animals treated with the vehicle served as negative control. Edema was induced by injecting 0.02 ml of 2.5% carrageenan in sterile saline into the plantar surface of the right hind paw. The difference of volumes between the basal and sequential measurements in the right hind paw was calculated as the edema formation. The paw volume was measured in an Ugo Basile plethysmometer (Comerio VA, Italy), at 0.5, 1, 2, 3, 4, 6, 8, 24 and 48 h.

### 3.5. Statistical analysis

The results are expressed as mean ± SEM (*n* = 6). Statistical significance was determined by analysis of variance and subsequent Duncan’s multiple range test (*p* < 0.05). The analysis was performed using Statistical Package for Social Sciences – SPSS statistical software (Chicago, IL, USA).

## 4. Conclusions

In conclusion, our data suggests that COS are able to induce anti-inflammatory effect mediated by cyclooxygenase inhibition and consequent reduction of prostaglandins. In addition, the efficacy of high-dose COS3 (500 mg/kg b.w.) was comparable to that of indomethacin, but during a shorter period. A single dose of 500 mg/kg b.w. may indeed be suitable to treat acute inflammation cases; however, further studies are needed to ascertain the effect upon longer inflammation periods as well as studies upon the bioavailability of these compounds.
